# Comparison of outcomes between off-pump versus on-pump coronary artery bypass surgery in elderly patients: a meta-analysis

**DOI:** 10.1590/1414-431X20165711

**Published:** 2017-03-02

**Authors:** Z.G. Zhu, W. Xiong, J.L. Ding, J. Chen, Y. Li, J.L. Zhou, J.J. Xu

**Affiliations:** 1 Department of Cardiothoracic Surgery, the Second Affiliated Hospital of Nanchang University, Nanchang, China; 2 Guangxi University of Chinese Medicine, Nanning, China; 3 Department of Gastroenterology, the Second Affiliated Hospital of Nanchang University, Nanchang, China

**Keywords:** Coronary artery bypass grafting/CABG, Off-pump, On-pump, Elderly, Meta-analysis

## Abstract

The aim of this study was to analyze if off-pump coronary artery bypass surgery (CABG) is associated with better treatment outcomes in elderly patients (>70 years of age) than on-pump CABG, using meta-analysis. Medline, PubMed, Cochrane and Google Scholar databases were searched until September 13, 2016. Sensitivity and quality assessment were performed. Twenty-two studies, three randomized control trials (RCTs) and 20 non-RCTs were included with 24,127 patients. The risk of death associated with on-pump or off-pump CABG in the RCTs were similar (pooled OR=0.945, 95%CI=0.652 to 1.371, P=0.766). However, in the non-RCTs, mortality risk was lower in patients treated with off-pump CABG than on-pump CABG (pooled OR=0.631, 95%CI=0.587 to 0.944, P=0.003). No differences were observed between the two treatment groups in terms of the occurrence of 30-day post-operative stroke or myocardial infarction (P≥0.147). In the non-RCTs, off-pump CABG treatment was associated with a shorter length of hospital stay (pooled standardized difference in means=-0.401, 95%CI=-0.621 to -0.181, P≤0.001). The meta-analysis with pooled data from non-RCTs, but not RCTs, found that mortality was lower with off-pump compared with on-pump CABG, and suggested that there may be some benefit of off-pump CABG compared with on-pump CABG in the risk of mortality and length of hospital stay.

## Introduction

Cardiac surgery is challenging in elderly patients. Most elderly patients have comorbidities that increase the risk of death due to coronary revascularization. The presence of comorbidities also may affect the incidence of overall postoperative complications, which can result in increased length of hospital stay and cost (1). Due to the increase of the aging population and of life expectancy in many countries, incidence of coronary artery disease due to atherosclerosis and the rate of surgical revascularization have increased (2). Coronary artery bypass surgery (CABG) is considered a safe treatment option in some high-risk patients (3). However, elderly patients are considered of high risk for surgery; advanced age is an independent predictor for mortality, stroke, renal failure, and atrial fibrillation following CABG (2,4,5).

There is an ongoing debate regarding the benefit of CABG with (on-pump) or without (off-pump) cardiopulmonary bypass surgery, particularly with respect to benefits in the elderly. Two prior meta-analyses have compared CABG-related adverse events in elderly patients (≥70 years of age (1) and octogenarians (6) between off-pump and on-pump CABG (1,6). In both studies, off-pump CABG was associated with a lower risk of stroke compared with on-pump CABG. However, the studies’ findings differed with regard to the benefit of off-pump CABG on the incidence of death and atrial fibrillation following surgery. The purpose of the current meta-analysis was to compare the clinical outcomes of on-pump and off-pump CABG in patients who were ≥70 years of age.

Although the UN/WHO definition of elderly is people ≥60 years of age (7), an important aim of this meta-analysis was to update the information of a prior meta-analysis performed a decade ago by Panesar et al. (1), who defined elderly as people ≥70 years old. Hence, we chose the same definition as the prior paper to make the findings comparable.

## Material and Methods

### Search strategy

This study was performed in accordance with the PRISMA guidelines. Medline, PubMed, Cochrane and Google Scholar databases were searched until September 13, 2016 using the following search terms: coronary artery bypass grafting/CABG, off-pump, on-pump, and elderly. Included studies were randomized controlled trials (RCTs), prospective two-armed studies, or retrospective studies that compared on-pump versus off-pump CABG in elderly patients, aged ≥70 years, and reported quantitatively the outcomes of interest. Letters, comments, editorials, case reports, proceedings, and personal communications were excluded. Studies that evaluated repeated CABG were also excluded. The list of relevant studies was hand-searched by two independent reviewers and if there was disagreement on study inclusion, a third reviewer was consulted.

### Data extraction and quality assessment

The following information/data were extracted from studies that met the inclusion criteria: the name of the first author, year of publication, study design, number of participants in each group, participant’s age and gender, and the major outcomes. The quality of the included studies was evaluated using the Hayden’s tool (7), which evaluates prognosis studies with regard to six areas of potential study biases: study participation, study attrition, measurement of prognostic factors, measurement of and controlling for confounding variables, measurement of outcomes, and analysis approaches. Quality assessment was also performed by two independent reviewers and a third reviewer was consulted to resolve any uncertainties.

### Statistical analysis

The primary outcome was overall mortality and secondary outcomes were stroke within 30 days of the CABG surgery, rate of myocardial infarction, and length of hospital stay. Odds ratio (OR) was used to evaluate the effect size for mortality, stroke, and myocardial infarction; an OR <1 indicates that off-pump CABG treatment was associated with lower risk of death, stroke, or myocardial infarction. Length of hospital stay is presented by standardized difference in means; negative values indicate shorter hospital stay in the off-pump group. Pooled estimate for odds ratio and standardized difference in means were calculated by DerSimonian and Laird random-effects model. A two-sided P-value <0.05 was considered to be statistically significant.

Heterogeneity was assessed using the Cochran Q and the I^2^ statistic. For the Q statistic, P<0.10 was considered to be statistically significant for heterogeneity. The I^2^ statistic indicates the percentage of the observed between-study variability due to heterogeneity. The suggested ranges are as follows: no heterogeneity (I^2^=0-25%), moderate heterogeneity (I^2^=26-50%), large heterogeneity (I^2^=51-75%), and extreme heterogeneity (I^2^=76-100%). Sensitivity analysis was carried out for the primary outcomes using the leave-one-out approach. Publication bias was assessed by constructing a funnel plot for the primary outcome. The absence of publication bias was indicated by the data points forming a symmetric funnel-shaped distribution. Egger’s test was performed to examine the symmetry of funnel plot; a one-tailed P>0.05 indicated there was no publication bias. Following recommendations from the Cochrane handbook (8), RCTs and non-randomized studies (non-RCTs) were analyzed separately. All analyses were performed using Comprehensive Meta-Analysis statistical software, version 2.0 (Biostat, USA).

## Results

### Search results

**Figure 1:**
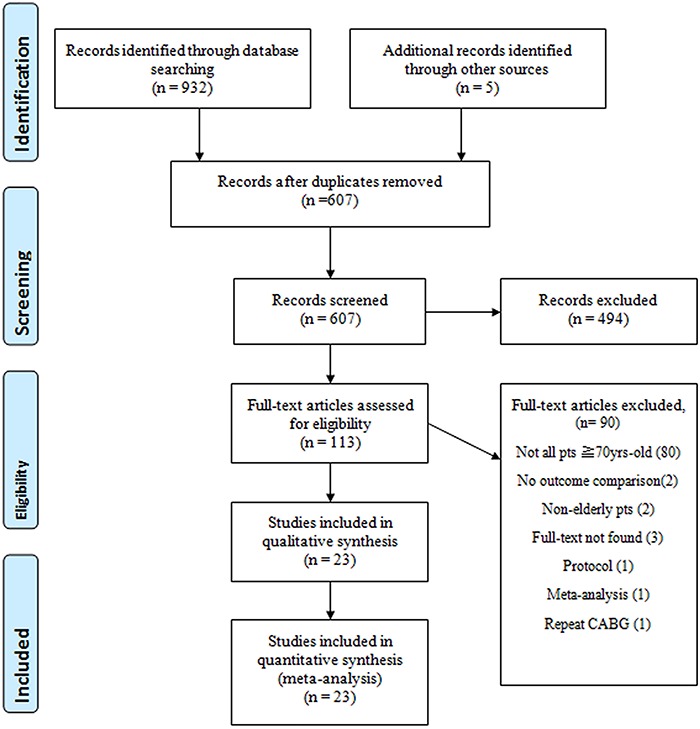
PRISMA flow diagram.

After removal of duplications, 607 of the 932 originally identified studies were screened for inclusion (Figure 1). Of these, 494 were excluded for not being relevant and an additional 90 were excluded for not having patients who were ≥70 years of age, not reporting findings that compared on-pump versus off-pump CABG, the complete text was not available, or the publication described only the study protocol.

Twenty-three studies were included in the meta-analysis: three RCTs (9–11) and 20 non-RCTs (Supplementary Table S1) (12–30). The number of patients ranged from 29 to 12,697 with 24,127 patients in total. The mean or median age ranged from 73.9 to 84.0 years. Except for one study (13 [Bibr B14]), all studies recruited mostly males, which ranged from 52 to 89%. The presence of hypertension, type 2 diabetes, and chronic obstructive pulmonary disease (COPD) varied across studies, as did renal function. The frequency of smokers also was heterogeneous among the studies. The length of follow-up in the studies ranged from 30 days to 10 years.

### Meta-analysis

**Figure 2:**
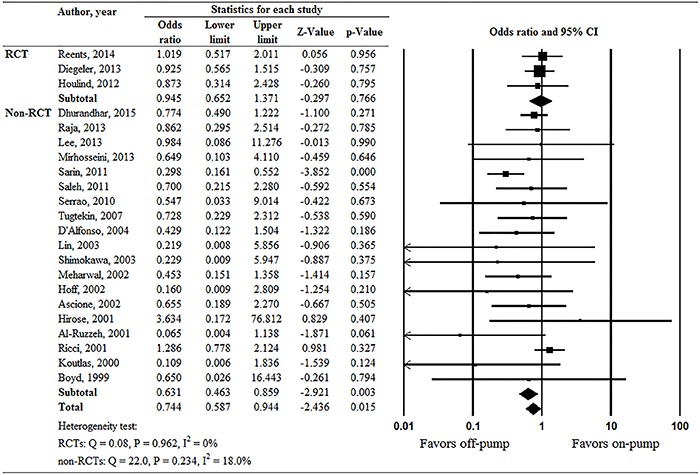
Summary table and forest plot of included studies for treatment effect of off-pump coronary artery bypass surgery (CABG) on mortality compared with on-pump CABG. RCT: randomized clinical trial.

All studies reported data on mortality except for one non-RCT (9). There was no evidence of heterogeneity among RCTs (Q=0.08 P=0.962, I^2^=0%) or among the non-RCTs (Q=22.0, P=0.234, I^2^=18.0%) in the mortality data across the studies. The pooled OR for the three RCTs indicated no increase in the risk of death between off-pump and on-pump CABG (pooled OR=0.945, 95%CI=0.652 to 1.371, P=0.766). In contrast, among the 19 non-RCTs, patients treated with off-pump CABG had a lower risk of death than those treated with on-pump CABG (pooled OR=0.631, 95%CI=0.587 to 0.944, P=0.003; Figure 2).

**Figure 3:**
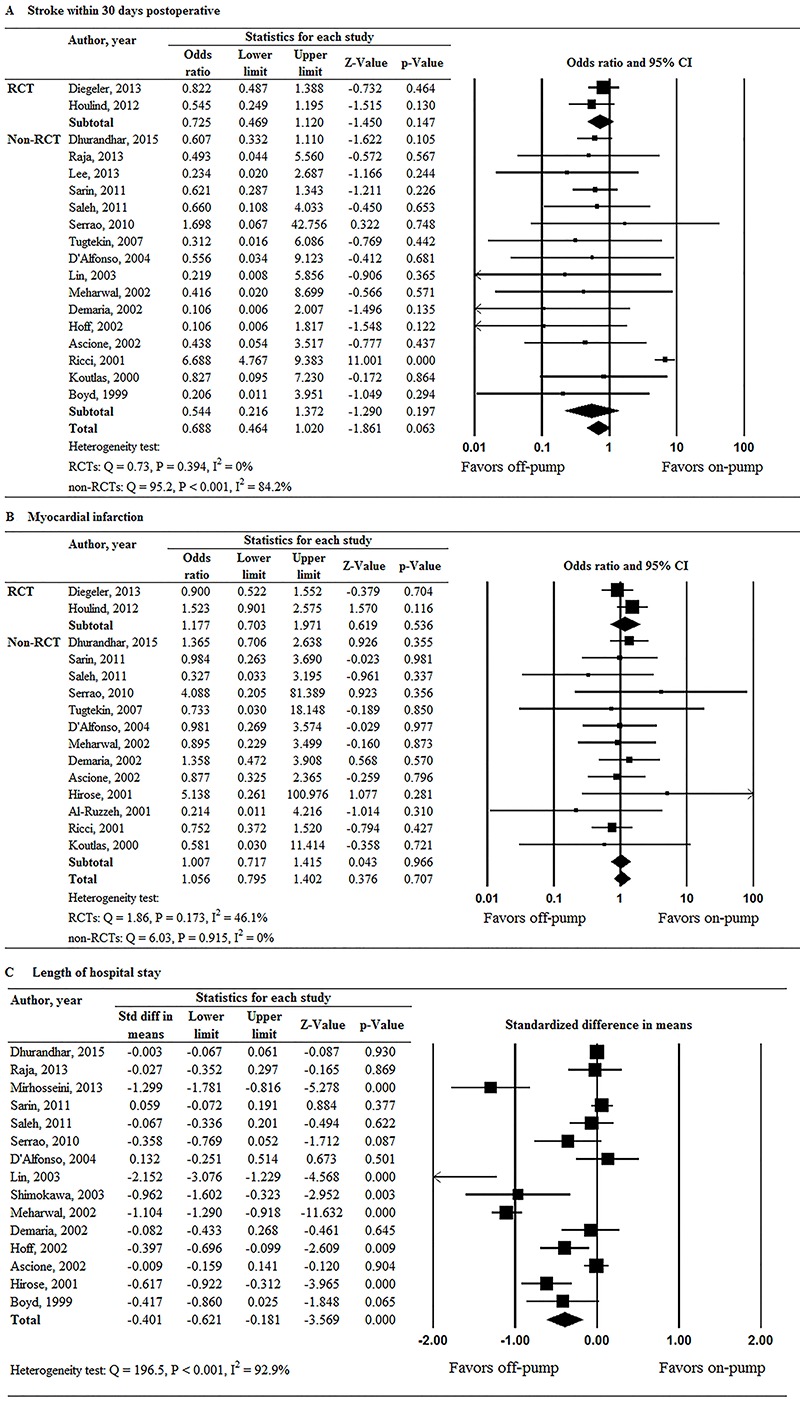
Summary tables and forest plots for treatment effect of off-pump and on-pump coronary artery bypass surgery (CABG) on (*A*) stroke within 30 days postoperative, (*B*) myocardial infarction, and (*C*) length of hospital stay. RCT: randomized clinical trial.

Eighteen studies reported data on stroke occurrence. There was evidence of extreme heterogeneity among the thirteen non-RCT studies for the occurrence of stroke between the two treatment groups (Q=95.2, P<0.001, I^2^=84.2%) but not for the two RCTs (Q=0.73, P=0.394. I^2^=0%). Regardless of study design, there was no difference in the risk of stroke occurring within 30 days post-operative between the off-pump and on-pump groups (RCTs: pooled OR=0.725, 95%CI=0.469 to 1.120, P=0.147; non-RCTs: pooled OR=0.544, 95%CI=0.216 to 1.372, P=0.197; Figure 3A). Similarly, there was no difference between the two treatment groups in the chance of myocardial infarction (RCTs: pooled OR=1.177, 95%CI=0.703 to 1.971, P=0.536; non-RCTs: pooled OR=1.007, 95%CI=0.717 to 1.415, P=0.966; Figure 3B).

Because only one RCT provided data on length of hospital stay, the meta-analysis was performed using data only from 15 non-RCTs. There was extreme heterogeneity across the studies (Q=196.5, P<0.001, I^2^=92.9%). Patients treated with off-pump CABG had shorter hospital stay than those with on-pump CABG (pooled standardized difference in means=-0.401, 95%CI=-0.621 to -0.181, P<0.001; Figure 3C).

### Sensitivity analysis

**Figure 4:**
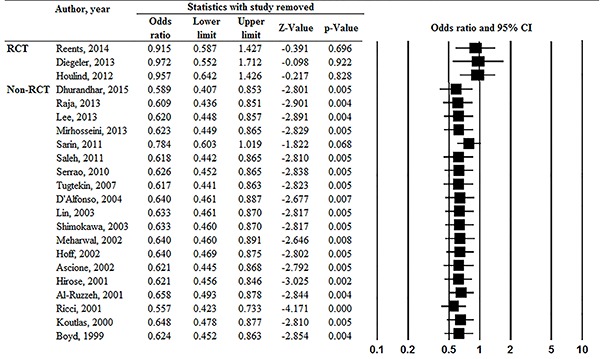
Sensitivity-analysis for treatment effect of off-pump coronary artery bypass surgery (CABG) on mortality compared with on-pump CABG.

Sensitivity analysis was performed using the leave-one-out approach. For the three RCTs, removal of any one study did not significantly influence the results indicating no individual study overly influenced the findings (Figure 4). In contrast, for the non-RCTs, removal of the study by Sarin et al. (15 [Bibr B16] [Bibr B17] [Bibr B18] [Bibr B19]) substantially affected the pooled odds (Figure 4).

### Publication bias and quality assessment

**Figure 5:**
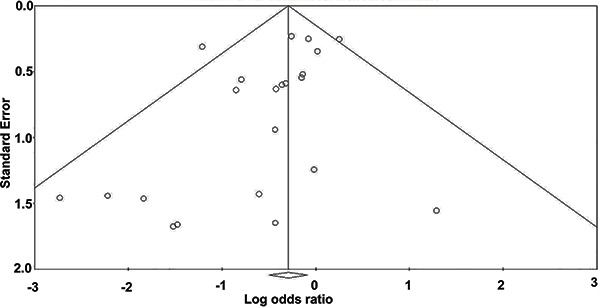
Funnel plot for publication bias.

**Figure 6:**
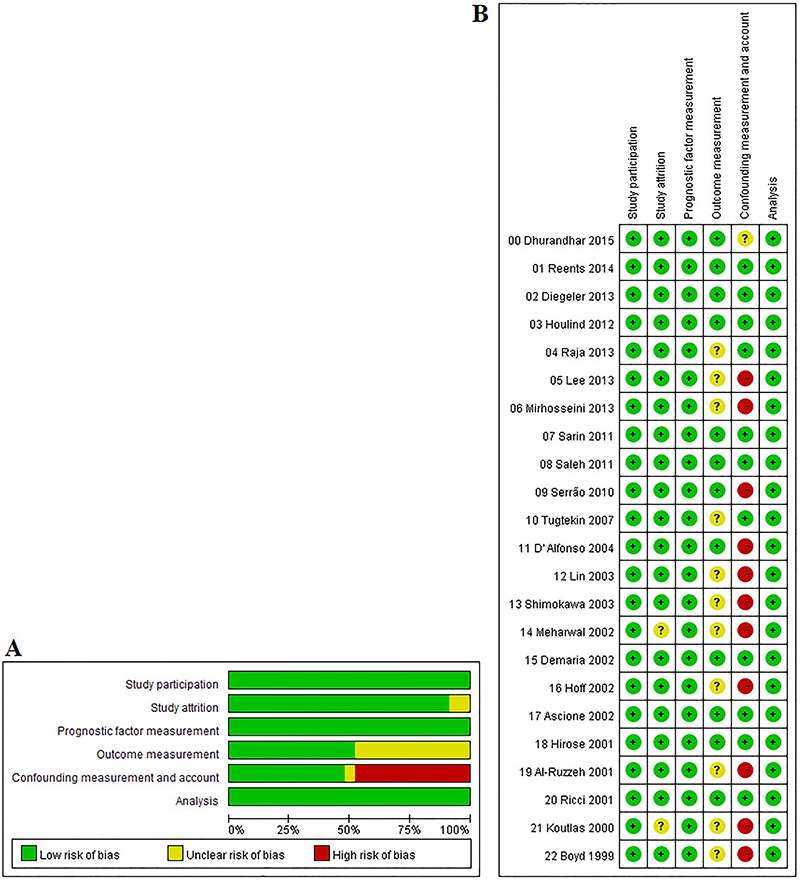
Quality assessment of included studies. *A*, Risk of bias assessment, and *B*, individual study assessment.

Results of the Egger’s test showed evidence of publication bias for the findings regarding overall mortality (t=1.90, P=0.036); beneficial treatment effect of off-pump CABG was found in studies with small sample size (Figure 5). Quality evaluation of the included studies showed overall adequate quality (Figure 6). About 50% of selected studies did not describe measurement of confounding factors and/or did not take into account confounding effects in the statistical analyses.

## Discussion

Due to the increase in the elderly population, the mean age of patients receiving CABG is rising (31); more than 80% of octogenarians have cardiovascular disease (32). Elderly patients are considered to be at significant risk of complications and death due to the presence of comorbidities (31). This study compared outcomes of off-pump and on-pump CABG in elderly adults who were ≥70 years of age. Twenty-three studies, three RCTs and 20 non-RCTs, with 12,697 patients were included. We found no difference in the risk of mortality between patients treated with on-pump or off-pump CABG in the RCTs. However, there was a reduced chance of death in patients who received off-pump CABG compared with on-pump CABG in the non-RCT studies. The difference in findings between the RCTs and the non-RCTs may be because patients included in the RCTs have lower operative risk due to enrollment criteria than those in the non-RCTs, which may be necessary for randomization into an experimental and control arm (1). Also, patients with more serious conditions may prefer the surgical method that their physician is more comfortable with or which has the higher success rate at their specific institution. The difference in findings between the RCTs and non-RCTs may also be due to the disproportionate number of publications available between study designs (3 *vs* 20, respectively) for each procedure, as well as the large heterogeneity in the data across studies.

The two treatment groups were similar in terms of the occurrence of 30-day post-operative stroke or myocardial infarction, suggesting that off-pump CABG is at least as effective as on-pump CABG. The findings from the prospective studies found that off-pump CABG treatment was associated with a shorter length of hospital stay. However, these findings may be confounded by the extreme heterogeneity of the data across the studies. Sensitivity analysis indicated that only one study (15) may have overly influenced the mortality findings for the non-RCT analysis. No evidence of publication bias was observed. Although, our findings differed with respect to mortality between RCTs and non-RCTSs and only non-RCTs were utilized to evaluate length of hospital stay, our findings suggest that there may be a benefit for off-pump compared with on-pump CABG in the risk of mortality and length of hospital stay in elderly patients.

Two prior meta-analyses have evaluated the use of off-pump and on-pump CABG in elderly patients (1,6). Panesar et al. (1) assessed early outcomes, in patients ≥70 years of age (n=4,921) who underwent either off-pump or on-pump CABG. Altarabsheh et al. (6) compared early adverse event following off-pump and on-pump CABG in octogenarians (n=18,310). Both our study and that of Panesar et al. (1) found a lower risk of death in the off-pump group then in the on-pump group. In the Panesar et al. study, a decrease in mortality risk associated with off-pump CABG was also observed in octogenarians. In contrast, Altarabsheh et al. (6) found that in octogenarians the risk of mortality was similar between CABG groups. Our study was similar to that of Panesar et al. who reported that the off-pump CABG group was associated with a shorter length of hospital stay than the on-pump CABG group. Both our study and that of Altarabsheh et al. found that the chance of myocardial infarction was comparable between treatment groups. Panesar et al. did not assess myocardial infarction. In contrast to our study, both Panesar et al. and Altarabsheh et al. found that the risk of stroke was lower in the off-pump CABG group compared with the on-pump group. Our finding with regard to stroke is limited by the extreme heterogeneity of the data across the studies. Both Panesar et al. and Altarabsheh et al. found no difference between groups in the rate of renal failure. We did not assess renal failure.

The inconsistencies in findings between the meta-analyses likely reflect the difference in number and designs of the included studies. In addition, the study of Altarabsheh et al. (6) focused on octogenarians while Panesar et al. (1) and our studies included patients that were ≥70 years of age. Moreover, the study of Panesar et al., which had a similar age population as ours, was performed 10 years ago, and since then there has been significant changes in both surgical equipment, physician technique, and medications that may have impacted the findings.

The heterogeneity we observed in the included studies may result in part from differences in patient populations, skills of the physicians, surgical procedures, and difference in patient inclusions/exclusion criteria, not only between RCTs and non-RCTs, but also across all studies. We did not compare the inclusion/exclusion criteria among the studies. In addition, not all the studies adjusted their data with propensity score matching analysis or multivariable analysis. For example, the RCT by Diegeler et al. (10) used multivariate models that incorporated clinical predictors to estimate the operative mortality, whereas the non-RCT study by Lin et al. (20 [Bibr B21] [Bibr B22]) reported data based on unadjusted variables. Some non-RCTs, such as by Sarin et al. (15), provided propensity-adjusted, retrospective review of their patient data. These issues are consistent with our quality assessment of the studies. Overall, there was about 50% risk of bias for the presence of confounding measurement. The discrepancies across these studies highlight the need for additional well-controlled studies that evaluate the use of off-pump and on-pump CABG in elderly patients.

Two other prior meta-analyses (33,34 [Bibr B35]) also evaluated the use of off-pump CABG in cardiovascular surgery, but in contrast to our study, they did not limit their analysis to patients who were ≥70 years of age. The meta-analysis of Sa et al. (34) included 47 RCTs with 13,524 patients (6,758 for off-pump and 6,766 for on-pump CABG). They found no difference between treatments in 30-day mortality or myocardial infarction. However, there was a difference between procedures in stroke favoring off-pump CABG (P=0.049). Godinho et al. (33) included nine randomized studies with 75,086 patients. They found an 18% reduction in mortality and a 25% lower risk of stroke with off-pump CABG compared with on-pump CABG (P≤0.03). A significant difference between the two surgical techniques with respect to procedure-associated complications, particularly kidney complications (P=0.74) and sepsis (P=0.93) was also found. The difference in findings between these two meta-analysis likely reflects the different studies included. Neither study evaluated differences in length of hospital stay between surgical techniques.

There are several limitations to our study. Extreme heterogeneity across the studies was observed both for 30-day post-operative stroke (I^2^=80.6%) and length of hospital stay (I^2^=92.2%). This type of variability between studies may compromise the reliability of statistical analysis. Although off-pump CABG was associated with lower mortality risk, the large variance in length of follow-up time from 30 days to 10 years may confound the findings. The effect of a treatment on a person’s health may evolve over time. Many of the complications may not occur until later in life, or the impact of treatment may not be obvious during the first few weeks after surgery, such that outcomes measured using longer follow-up times may be more reliable than shorter follow-up times. The definition of secondary endpoints of myocardial infarction and stroke, when reported, varied across studies, which may have impacted the results; only one RCT (11) and four non-RCTs (23 [Bibr B24],^25^ [Bibr B26] [Bibr B27],^28^,^29^) describe a definition for myocardial infraction and/or stroke. In addition, some of the non-RCTs used in our study may have included selection bias, which may confound the findings. For surgical techniques, such as the ones investigated, randomization is sometimes difficult and unethical. Physician skills, health condition of individual patients, the availability of equipment, and appropriate supportive staff are all potential confounding factors that can affect the choice of surgery. Patients having surgery must give their informed consent to the procedure for ethical reasons, which may also cause bias and make it difficult to blind a study. Although our quality assessment found a low risk of bias overall in study participation and attrition, it is unlikely that the study results are highly affected by selection bias. This may explain the lack of randomized control trials that are available currently.

In summary, our study and that of Panesar et al. (1) suggest that off-pump CABG may reduce the risk of mortality in elderly patients who are ≥70 years of age with ischemic heart disease compared with conventional CABG. However, in our study, the findings for survival benefit were only observed using the pooled data from the non-RCTs, and not from the RCTs. Although RCTs are the “gold standard” for clinical studies, data from non-RTCs studies should not be dismissed as they have potential clinical significance. More randomized trials are needed in order to explore further the benefits of using off-pump CABG compared to conventional CABG in elderly patients and to determine if it is a safe surgical option for high-risk patients with comorbidities, which often increase the rate of morbidity or death following cardiopulmonary bypass surgery.

## Supplementary Material

Click here to view [http://bjournal.com.br/supplementary_material/5711.pdf].
